# The Bi-Chromate Battery

**Published:** 1883-09

**Authors:** 


### The Bi-Chromate Battery.

M. Trouve is stated to have considerably improved the bichromate battery in the matter of permanency, by super-saturating the liquid. He takes 150 grammes of bi-chromate of potash powder to a litre of water, and after shaking, adds, drop by drop, 450 grammes of sulphuric acid. The liquid warms and the salt dissolves, while no crystals are formed on cooling, nor are chrome alum crystals deposited in the cell. The elements are arranged with two carbons to each zinc, the latter being so placed that it can be withdrawn from the solution. With twelve elements and the solution above described, it is stated that ten incandescent lamps can be kept at work for five hours, each lamp going ten candles. The E. M. F. of the cell is stated to be 2 volts with fresh solution.

